# The association between county ordinances allowing off-road vehicles on public roads and crash rates

**DOI:** 10.1186/s40621-024-00516-2

**Published:** 2024-07-18

**Authors:** Christopher D. Monson, J. Priyanka Vakkalanka, Gerene M. Denning, Nicholas R. Stange, Charles A. Jennissen

**Affiliations:** 1 Division of Critical Care, Department of Pediatrics, University of Rochester, 601 Elmwood Ave, Box 667, Rochester, NY USA; 2 Department of Emergency Medicine, Roy J. and Lucille A. Carver College of Medicine, University of Iowa, Iowa City, IA USA; 3 Department of Epidemiology, College of Public Health, University of Iowa, 200 Hawkins Drive, Iowa City, IA 52242 USA; 4 Saint Louis University School of Medicine, Saint Louis University, 1402 S Grand Blvd, St. Louis, MO 63104 USA; 5 Stead Family Department of Pediatrics, Roy J. and Lucille A. Carver College of Medicine, University of Iowa, Iowa City, IA USA

**Keywords:** All-terrain vehicles, Collisions, Crashes, Legislation, Off-highway vehicle, Off-road vehicles, Ordinance, Recreational off-highway vehicles, Roads, Safety, Utility task vehicles

## Abstract

**Background:**

Legislative bodies across the country have increasingly allowed off-road vehicles (ORVs) including all-terrain vehicles (ATVs) and utility task vehicles (UTVs) on public roads, an environment for which they are not designed. In 2004, Iowa gave individual counties the discretion to pass ordinances allowing ORVs on public roadways. The objective of this study was to evaluate the relationship between the passage of ORV ordinances and ORV crash rates, especially on public roads.

**Methods:**

An Iowa ORV roadway ordinance database and an Iowa ORV crash database (2002–2018) for all 99 counties were compiled. Crashes for which county location could not be determined were excluded. Utilizing a zero-inflated Poisson model, correcting for background crash frequency trends and population, investigators compared the relative rates of crashes after ordinance passage to time points before ordinance implementation and to counties without such ordinances. Sub-analyses, including that focused on more recent years (2008–2018), were also performed.

**Results:**

Forty-five county ORV roadway ordinances went into effect between 2011 and 2018 and 2,347 crashes (69%) met inclusion criteria. Adjusted for year, there was a 58% greater ORV crash rate in counties after passing an ORV roadway ordinance (incidence rate ratio (IRR) 1.58, 95% CI 1.32–1.90). Roadway crashes (n = 834) increased 48% after ordinance passage (IRR 1.48, 95% CI 1.14–1.94). This roadway crash association remained statistically significant when analysis was limited to the years 2008–2018 (IRR 1.39, CI 1.06–1.83, n = 544); to ATV crashes only (IRR 1.70, CI 1.20–2.40, n = 683); and to ATV crashes excluding counties with UTV-only ordinances (IRR 1.74, CI 1.40–2.15, n = 2,011).

**Conclusions:**

ORV roadway and total crashes increased significantly after implementation of county ordinances allowing ORVs on public roadways and when compared to counties without such ordinances. It is likely that these increased crashes have resulted in more injuries and possibly deaths. Results from this study may help inform policymakers as they consider legislation regarding ORV usage on public roads.

## Background

Off-road vehicles (ORVs) account for over 700 deaths and more than 100,000 emergency department visits annually (Topping 2021; Zhang 2023). Despite safety concerns, they remain popular for both occupational and recreational purposes. ORVs include all-terrain vehicles (ATVs) and side-by-side vehicles which are often referred to as utility task vehicles (UTVs) or recreational off-highway vehicles (ROVs). For the purpose of this manuscript, we will refer to all side-by-side vehicles as UTVs. All of these vehicles are designed to be used off-road only and at relatively lower speeds than roadway vehicles (Specialty Vehicle Institute of America 2017; Jennissen et al. 2022a, b; United States Government Accountability Office 2010; Denning and Jennissen 2016). Manufacturers explicitly state that ATVs are not designed for roadway use (Denning and Jennissen 2016; Polaris Inc 2022; Yamaha Motorsports 2022; Bombardier Recreational Products, Inc 2021), and the U.S. Consumer Product Safety Commission (CPSC) requires consumer warning labels regarding this danger (Code of Federal Regulations 2008).

Prior studies have shown that ORV deaths and severe injuries are more common on roadways than off (Denning and Jennissen 2016; Denning et al. 2013a, 2013b; Williams et al. 2014; Weintraub and Best 2014; Rodgers 1999, 2008; Rodgers and Adler 2001; Jennissen et al. 2024). Over 60% of ATV-related fatalities in the United States have occurred on public roads (Denning et al. 2013a). Although paved roadways may pose additional risks than those unpaved, riding ATVs on either involves significantly greater risks than riding off-road (Denning and Jennissen 2016). Although the likelihood of a collision with another motor vehicle is much higher when riding on public roads, over two-thirds of ATV-related roadway fatalities and injuries do not involve another vehicle; they are single vehicle crashes (Denning and Jennissen 2016; Denning et al. 2013a, 2013b). The vehicle’s off-road design is a significant contributor to this. Unsafe riding behaviors, such as alcohol use, multiple riders, and decreased helmet use, have also been found to be common and more likely on public roads (Denning and Jennissen 2016; Denning et al. 2013a, 2013b; Williams et al. 2014). These findings suggest that driving on roadways is among the most dangerous practices by ORV riders and accordingly a significant public health concern.

ORV regulations are not standardized and vary considerably among states, counties, and municipalities. In recent years, legislative bodies across the country have increasingly passed laws allowing ATVs and UTVs access to public roads for recreation and transportation purposes (Weintraub and Best 2014). The impact of this legislation on public health safety is unknown. While some states have legalized ORV on-road use statewide, the Iowa state legislature gave individual counties and municipalities the discretion to pass ordinances allowing ATVs/UTVs on roadways in 2004. Over the subsequent 17 years, a significant proportion of counties enacted such legislation. This created a unique opportunity to compare similar populations over time, some of which had these ordinances in place and others that did not. The objective of this study was to evaluate the relationship between the passage of ORV ordinances and ORV crash rates, especially on public roads.

## Methods

### Statewide ORV crash database

This was a retrospective, quasi-experimental study evaluating the impact of ordinance passage on county-level crash rates utilizing anonymized epidemiological data. A previously compiled statewide ORV crash database composed of records from the Iowa Department of Transportation (DOT), Department of Natural Resources (DNR) and State Trauma Registry (STR) from 2002 to 2018 was updated for this study. The University of Iowa Institutional Review Board approved both the initial creation of this database and the usage of the database for this study. Detailed descriptions of this database can be found in previously published studies (Denning et al. 2013a, 2013c; Qin et al. 2017; Jennissen et al. 2021).

Separate datasets were cross-referenced to eliminate duplicates and codify information previously lost to descriptive text fields so as to maximize inclusion of crashes from the broader database in this study. To be included in the final dataset, crashes must have occurred in the state of Iowa, and must have had occurrence month, year, and county location documented. In addition, crashes had to have on/off public road status and specific ORV style (ATV/UTV) documented to be included in related sub-analyses. Crashes not meeting either or both of the latter criteria for sub-analyses were still used in the broader analyses.

### Iowa ORV roadway ordinance database

A novel ORV roadway ordinance database from 2004 to 2021 was compiled for all 99 Iowa counties including ordinance text, scope and effective date. Due to the decentralized nature of county legislation and recordkeeping, a manual county-by-county survey was completed to determine whether a relevant ATV and/or UTV roadway ordinance had been passed. If an ordinance existed, then the date the ordinance went into effect and the full text of the ordinance were collected. It should be noted that these ordinances did not include off-road vehicles with two wheels (i.e., dirt bikes). Data were obtained via a variety of methods. Initial search was performed via web search engines using combinations of the following terms: Iowa, county, ATV, UTV, OHV, ORV, all-terrain, utility, off-highway, off-road, vehicle, roadway, ordinance, law, board of supervisors. In some cases, counties had searchable ordinance codes or online record of the minutes or transcripts from board of supervisors’ meetings. Other counties only had paper records, so direct contact with county auditors via email or phone was required.

### Statistical analysis

Crashes were aggregated to evaluate a monthly crash rate for each county, which was then adjusted for county population. Since crash events are rare and there were often months where counties had no crashes, we evaluated the incident rate ratio (IRR) using a zero-inflated Poisson model, adjusted for calendar year (Yang 2015). In the primary analysis, the IRR was compared between counties that had an ordinance established during the month of the crash and those which did not. For our secondary outcomes, we evaluated the IRR for crashes of ATVs only, crashes only on public roads, and ATV crashes on public roads. A sensitivity analysis for each model was included by limiting the timeframe to more recent years (2008–2018) to account for variation over time. As some counties passed legislation only related to UTVs, we conducted a sensitivity analysis excluding counties with only UTV legislation. Finally, to account for county-level variability in passing an ordinance, we additionally conducted a sensitivity analysis that examined the IRR in a subset of counties that ever passed an ordinance during the study period. All analyses were conducted using SAS 9.4 (Cary, North Carolina).

## Results

Forty-five county ORV roadway ordinances went into effect in the state of Iowa from 2011–2018 and were utilized in data analysis. An additional 27 counties passed ordinances after our study period through July 2021. The text of these ordinances was extremely similar, often copied word-for-word from a neighboring county. The only meaningful difference in ordinance content was that 6 of the 45 included in this study only pertained to UTVs (excluded ATVs); all other ordinances pertained to both ATVs and UTVs.

A total of 3,426 unique crashes were identified including 3,068 ATV crashes (90%), 222 UTV crashes (6%) and 136 crashes (4%) in which the vehicle type (ATV or UTV) could not be reliably determined. Of the total, 2,347 crashes (68%) met inclusion criteria (2,115 ATV; 180 UTV) with verifiable crash date and county location data. Of these, 1775 crashes (76%) had information regarding roadway/off-road occurrence with 834 crashes (47%) on public roads.

**Fig. 1:**
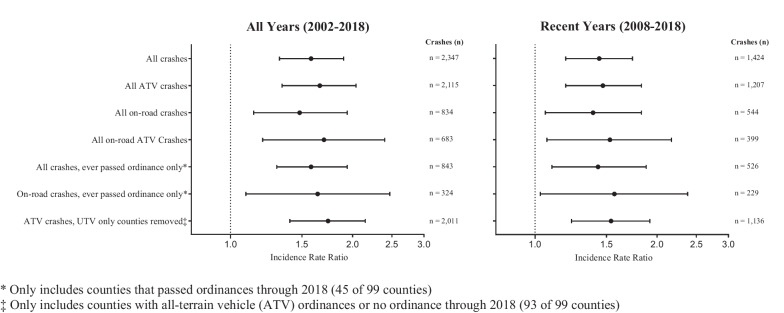
Off-road vehicle crash incident rate ratios. County off-road vehicle (ORV) crash incident rate ratios (IRR) after passage of ordinances allowing ORVs on public roadways as compared to before passage and to counties that did not pass such ordinances from 2002 to 2018 and sub-group analyses for the years 2008–2018

The ORV crash rate increased 58% in counties after passing an ORV roadway ordinance (incidence rate ratio (IRR) 1.58, 95% CI 1.32–1.90). See Table 1 and Fig. 1**.** Public roadway crashes increased 48% after ordinance passage (IRR 1.48, 95% CI 1.14–1.94). ATV crashes (not including UTVs) and roadway ATV crashes increased 66% (IRR 1.66, 95% CI 1.34–2.04) and 70% (IRR 1.70, 95% CI 1.20–2.40), respectively, after ORV ordinance passage as compared to crash rates prior to passage and to counties without ORV ordinances. Similarly significant effects were seen when looking only at crashes in more recent years (2008–2018) or only in counties that eventually passed ordinances within the study period (45 counties). When excluding counties that passed ordinances pertaining only to UTVs, ATV crashes increased 74% (IRR 1.74, 95% CI 1.40–2.15) in counties with ordinances allowing roadway use.

**Table 1:** County off-road vehicle (ORV) crash incident rate ratios (IRR) after passage of ordinances allowing ORVs on public roadways as compared to before passage and to counties that did not pass such ordinances

Data Subset Description	All Years (2002–2018)			Recent Years (2008–2018)		
	Crashes	IRR	95%CI	Crashes	IRR	95%CI
All crashes	2,347	1.58	1.32–1.90	1,424	1.44	1.19–1.74
All ATV crashes	2,115	1.66	1.34–2.04	1,207	1.47	1.19–1.83
All on-road crashes	834	1.48	1.14–1.94	544	1.39	1.06–1.83
All on-road ATV crashes	683	1.70	1.20–2.40	399	1.53	1.07–2.17
All crashes, ever passed ordinance only*	843	1.59	1.22–2.06	526	1.43	1.10–1.88
On-road crashes, ever passed ordinance only*	324	1.64	1.09–2.47	229	1.57	1.03–2.38
ATV crashes, UTV only counties removed‡	2,011	1.74	1.40–2.15	1,136	1.54	1.23–1.92

^*^ Only includes counties that passed ordinances through 2018 (45 of 99 counties)

^‡^ Only includes counties with ATV ordinances or no ordinance through 2018 (93 of 99 counties)

## Discussion

Studies have shown that roadway use of ORVs is clearly a dangerous practice (Denning and Jennissen 2016; Denning et al. 2013a, 2013b; Williams et al. 2014; Weintraub and Best 2014; Jennissen et al. 2024), but the impact of legislation allowing ORV use on public roads has not previously been quantified. Our study shows that legislation allowing the use of ORVs on public roadways was associated with significantly increased roadway and total crashes. Overall, we found a 58% increase in crashes in counties after permissive ORV roadway ordinance implementation as compared to before passage and as compared to counties that did not pass such ordinances. Based on this and extrapolating from the most recent 5 years of CPSC injury and death data from Iowa (Topping 2021; Zhang 2023), a 58% increase in ORV crashes could result in an estimated 776 additional emergency department visits and 5 additional deaths per year in the state of Iowa.

ORVs are designed for off-road use only (Specialty Vehicle Institute of America 2017; Jennissen et al. 2022a, b; Denning and Jennissen 2016). They have a relatively narrow track so they can travel between obstacles and a high clearance that allows them to drive over rough terrain. However, this gives ORVs a relatively high center of gravity that increases their risk of rollover. In addition, roadway vehicle tires have shallow tread designed to continually grip and release roadway surfaces. In contrast, ORVs have low-pressure tires with knobby tread that are designed to grab off-road terrain but can unevenly and unpredictably grab public roadway surfaces leading to loss of ORV control (Jennissen et al. 2022a, b; United States Government Accountability Office 2010; Denning and Jennissen 2016; Carman et al. 2010; Huhlein 1998). Roadway vehicles also have an open rear differential that allows the outer tires to travel faster than the inner tires, creating a tighter turning radius. ATVs and many UTVs have a solid rear axle or locked rear differential, and operators can easily misjudge the speed they need to slow down to in order to successfully negotiate a turn or curve (Jennissen et al. 2022a, b; United States Government Accountability Office 2010; Denning and Jennissen 2016; Carman et al. 2010; Huhlein 1998). Thus, ORVs have a number of off-road design features that increase the risk of loss of control and rollover, especially at the speeds often traveled on public roads.

In our study, many roadway ORV crashes were noted both in counties before permissive ordinance passage and in counties where usage remained illegal, suggesting inadequate enforcement of ORV roadway prohibition. Previous studies found limited and variable enforcement of restrictive ORV laws, thus limiting their potential to prevent deaths and injuries (Qin et al. 2024). If this legislation were better enforced, the potential impact of prohibitive ORV roadway legislation may be considerably greater than that seen in our study. Strategies are needed to support enforcement of restricted ORV roadway access and should be targeted toward jurisdictions that have these laws in place.

In the course of cataloguing ORV ordinance data for all 99 Iowa counties, the rationales for permissive roadway ordinances by supporters were occasionally noted in board of supervisor meeting transcripts. In some cases, county officials and members of the public cited a desire to travel between different farmland plots via connecting public roads for work. However, a longstanding state law already allowed the use of ORVs on public roadways for agricultural purposes. Despite the lack of data demonstrating economic benefit to opening up public roads to ORV use, individuals in some counties expressed concerns of potentially losing business to a neighboring county that had already passed an ordinance. This phenomenon was a likely contributor to the geographic spread of ordinances from a nidus county to adjacent counties throughout the study period.

As this study was being conducted in 2021–2022, the Iowa state legislature began circulating a bill to both legalize the use of ATVs/UTVs on county public roads statewide and to restrict the ability of county boards of supervisors to limit such usage (Reynolds 2022). Despite opposition by health experts, law enforcement organizations and even some off-highway vehicle associations (The Iowa Legislature 2022), the legislature passed House File 2130. In June of 2022, the bill was signed into law by the governor, thereby enacting the legislation investigated in this study across the entire state (Reynolds 2022).

### Limitations

Permissive OHV roadway laws, similar to that studied, have been enacted in other states or counties of other states. However, this study was performed in the state of Iowa and may not be generalizable to other states and counties. The sources for our crash database are robust but not all-inclusive, as crashes may have been unreported, may have lacked detailed vehicle categorization by state or healthcare officials resulting in omission from the crash database, and/or may have been excluded from this study due to lack of county-level location information.

As this was a quasi-experimental study, we were evaluating the impact of an exogenous event, i.e., the passing of ordinances, on county-level crash rates. Other factors may also have affected crash rates across time and location, though ordinance enactment remains the most plausible cause. One way we investigated the effect of location was to analyze the change in crash rate only in the subset of counties that passed an ordinance (excluding the crashes from counties that did not). These counties are generally more rural, with lower population density than counties that never passed ordinances. However, we found no skew of the results when limiting to these counties, with an almost identical 59% increase in crash rates on all surfaces and an even higher 64% increase in crash rate when looking at on-road only crashes in this subset of counties.

Regarding our calculations of the additional emergency department visits and deaths per year related to the passage of permissive ORV ordinances in the state of Iowa, these estimates assume relatively constant injury and death rates, which may change over time with changes in equipment design, use, and implementation of safety regulations. Similarly, crash rates seen in this study may change over time due to numerous factors and should be reassessed as the legislative, technological, and cultural landscape evolves.

## Conclusions

ORVs are widely used across the country and crashes remain a significant public health problem, especially on public roadways. Our study demonstrates that the passage of ordinances allowing ORVs on public roads is associated with a significant increase in ORV crashes. Such legislative actions have become more frequent across the nation and should be considered poor public health policy and promptly reversed. This study is currently the best evidence that restricting ORV use on public roads is a successful strategy in preventing ORV crashes and, consequently, ORV-related injuries and deaths.

## Data Availability

Data and materials are available to other parties for research purposes after a data sharing agreement plan is agreed to and signed. Those interested should contact the corresponding author.

## Abbreviations

ATV: All-terrain vehicle
CPSC: Consumer Product Safety Commission
DNR: Department of Natural Resources
DOT: Department of Transportation
IRR: Incident rate ratio
OHV: Off-highway vehicle
ORV: Off-road vehicle
ROV: Recreational off-highway vehicle
SAS: Statistical Analysis System
STR: State Trauma Registry
U.S.: United States
UTV: Utility task vehicle
